# Cancer Is a Major Determinant of Postoperative Atrial Fibrillation After Cardiac Surgery

**DOI:** 10.3390/jcm14062117

**Published:** 2025-03-20

**Authors:** Georgios P. Georghiou, Andrew Xanthopoulos, George Kanellopoulos, Panos Georghiou, Amalia Georgiou, John Skoularigis, Grigorios Giamouzis, Konstantinos Lampropoulos, Ioannis Patrikios, Filippos Triposkiadis

**Affiliations:** 1 Department of Cardiothoracic Surgery, Aretaeio Hospital, 2414 Nicosia, Cyprus; g.georghiou@aretaeio.com (G.P.G.); dr.george.kanellopoulos@gmail.com (G.K.); 2 Medical School, European University Cyprus, 2404 Nicosia, Cyprus; lampropouloskon@gmail.com (K.L.);; 3 Department of Surgery, Sackler Faculty of Medicine, Tel Aviv University, Tel Aviv 6997801, Israel; 4 Department of Cardiology, University General Hospital of Larissa, 41334 Larissa, Greece; andrewvxanth@gmail.com (A.X.); iskoular@gmail.com (J.S.);; 5 Queen Mary University of London, London EC1M 6BQ, UK; 6 Lithuanian University of Health Sciences, 44307 Kaunas, Lithuania; amalia.georgiou.med@gmail.com; 7 3rd Department of Cardiology, Euroclinic of Athens, 11521 Athens, Greece

**Keywords:** postoperative atrial fibrillation, cardiac surgery, cancer

## Abstract

**Background/Objectives:** Postoperative atrial fibrillation (POAF) occurs frequently after cardiac surgery and is associated with increased morbidity and mortality. The pathogenesis of POAF in this setting is complex and not completely understood. Since cancer is a well-known risk factor for AF, the aim of this study was to identify potential predictors, including cancer, of POAF after cardiac surgery. **Methods**: This prospective study included 400 consecutive patients in sinus rhythm who underwent elective cardiac surgery in Aretaeio Hospital (Nicosia, Cyprus) from January 2020 till January 2023. The primary outcome was the development of POAF during hospitalization, defined as any documented AF episode lasting >30 s. Predictors of the primary outcome were studied using univariable and multivariable logistic regression analysis. **Results**: Of the 400 patients (68 [61–73] years, 64 [16%] females) studied, 66 (16.5%) developed POAF. Among the variables examined, the only predictors of POAF were cardiopulmonary bypass time (odds ratio [OR] = 1.001, 95% confidence interval = [95% CI, 1.000–1.001], *p* = −0.031) and cancer (OR = 3.852, 95% CI = [1.535–9.664], *p* = 0.004). Cancer was present in 13 (4%) and in 10 (15%) of patients without and with POAF, respectively (*p* < 0.001). **Conclusions**: Cancer was associated with a dramatic increase in POAF risk early after elective cardiac surgery in this study. Whether patients developing POAF after cardiac surgery should be searched for cancer deserves further investigation.

## 1. Introduction

Postoperative atrial fibrillation (POAF) occurs in approximately 30% of cardiac surgical patients [1]. In the vast majority of the cases (approximately 90%) POAF appears during the first six days after cardiac surgery, a period coinciding with the peak of the postoperative systemic inflammation response [2]. Most POAF episodes are of short duration and usually resolve without intervention within days or weeks after cardiac surgery [3]. However, even short-lived POAF is associated with the prolongation of hospital stay, higher health care costs, and adverse long-term clinical outcomes such as stroke, heart failure, recurrent hospitalization, and death [4,5]. Unfortunately, since POAF usually converts to sinus rhythm spontaneously and usually does not cause hemodynamic compromise, its importance has often been undermined [6].

There is compelling evidence that AF is associated with cancer and vice versa. A recent meta-analysis of observational studies demonstrated an approximately 47% higher risk of AF in patients with cancer compared with patients without cancer [7]. Conversely, an increased risk of incident cancer has been reported in patients with known AF [8]. The bidirectional association between AF and cancer has been attributed to the sharing of common risk factors by these two conditions [9].

The number of patients with a history of cancer undergoing cardiac surgery has increased over time [10]. Yet, the currently used risk scores for the prediction of POAF do not include cancer and offer, at best, moderate prediction for POAF after cardiac surgery [11]. The purpose of this study was to identify potential predictors of POAF with special emphasis given to the impact of the presence of a history of cancer on POAF risk.

## 2. Materials and Methods

### 2.1. Patient Sample and Data Collection

This prospective clinical trial, which was performed by members of the Department of Cardiothoracic Surgery from Aretaeio Hospital (Nicosia, Cyprus), included four hundred (400) consecutive patients admitted between January 2020 and January 2023. Participants were adult patients (aged ≥ 18 years) in sinus rhythm (absence of palpitations and presence of sinus rhythm in two electrocardiograms the week prior to surgery) undergoing cardiac surgery for primary, elective interventions predominantly on the coronary arteries, the cardiac valves, or the ascending aorta, or a combination of these.

The primary outcome was the development of new-onset POAF during hospitalization. POAF is defined as any documented AF episode lasting >30 s recorded either by continuous telemetry throughout hospitalization or on a 12-lead electrocardiogram performed daily and when the patient reported experiencing symptoms. All patients had continuous telemetry monitoring at least during the first 48 h by an offsite central monitor unit, and once identified, every arrhythmia event was confirmed by a cardiologist.

Blood samples were taken preoperatively to determine hematological and biochemical markers. Hemoglobin (Hb) and red blood cell distribution width (RDW) were measured with the use of a Yumizen H 500 device (Horiba, Irvine, CA, USA), while urea, creatinine, and electrolytes (i.e., sodium and potassium) were measured with the use of a Cobas 8000 (Roche, Mannheim, Germany). The transthoracic echocardiography examinations were performed with the use of an HD11 XE device (Philips, Eindhoven, The Nederlands). The left ventricular ejection fraction (LVEF) was determined with the biplane “methods of discs” in accordance with the guidelines of the European Association of Cardiovascular Imaging [12]. Coexisting morbidities (valvular heart disease, heart failure, coronary artery disease, hypertension, cancer, chronic obstructive pulmonary disease, dyslipidemia, hyperthyroidism, chronic kidney disease, diabetes, mellitus, and obesity) and other patient information were obtained from patient charts and surgical reports, were registered, and were reviewed by two of the authors (G.G. and F.T.).

### 2.2. Statistical Analysis

Continuous variables are expressed as median (25th and 75th quartiles) and compared with the Mann–Whitney U test. Dichotomous variables are reported as absolute values and proportions and compared using the Wald test. Baseline variables that significantly differed between patients without and with POAF as well as the type of surgery (1 = coronary artery bypass grafting [CABG]; 2 = CABG plus valve surgery; 3 = valve surgery; 4 = aortic surgery; 5 = other) were initially evaluated with univariable logistic regression analysis with POAF as the dependent variable. Those that reached statistical significance were subsequently entered into a multivariable regression model to identify potentially independent predictors of POAF. For each variable, the odds ratios (ORs) along with the 95% confidence intervals (95%CI) are reported. A *p* value < 0.05 was considered statistically significant. The SPSS statistical package was used for data analysis (IBM Corp., released 2023, IBM SPSS Statistics for Windows, Version 29.0.2.0, IBM Corp, Armonk, NY, USA).

### 2.3. Ethical Considerations

This study was conducted in accordance with the Declaration of Helsinki and approved by the Ethics Committee of Aretaeio Hospital, Cyprus (Protocol No. 0001/2020, Date: 25 November 2020) and the Institutional Review Board (or Ethics Committee) of the University of Thessaly (Protocol No. 6619, Date: 20 November 2020). All patients provided written informed consent.

## 3. Results

**Figure 1:**
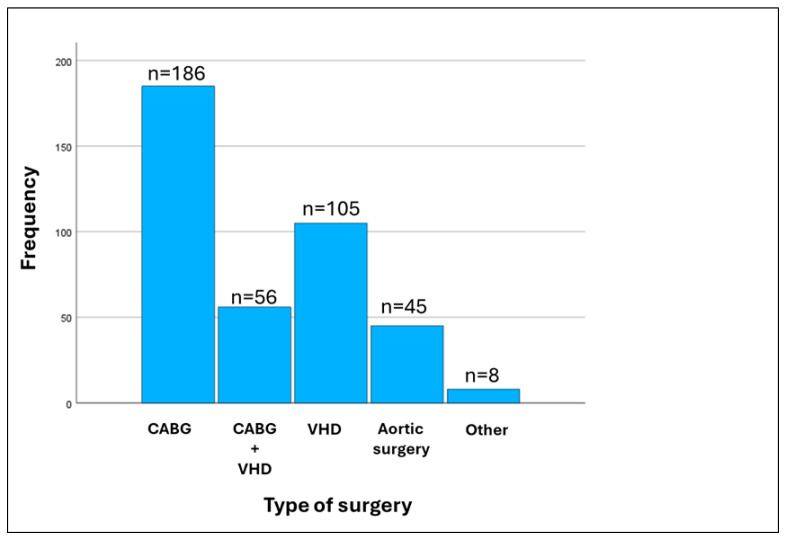
Histogram of the types of surgery performed in the study population. CABG, coronary artery bypass grafting; VHD, valvular heart disease; other, cardiac tumors and lead extraction.

The types and frequency of cardiac surgical procedures performed in the study patients are presented in Figure 1. Among the 400 (age 68 [61–73] years, 64 [16%] females) patients studied, 66 (16.5%) developed POAF. The baseline demographic, clinical, and laboratory characteristics, as well as the coexisting morbidities of the study patients classified by the absence or presence of POAF [POAF (−) and POAF (+), respectively], are presented in Table 1. The LVEF was lower in POAF (−) than in POAF (+) group (significantly higher than 50% in both groups), whereas the cardiopulmonary bypass time and the prevalence of valvular heart disease, cancer, and dyslipidemia were lower in the POAF (−) than in POAF (+) group. All the aforementioned factors were associated with the development of POAF in the univariable logistic regression analysis (Table 2). Likewise, the type of surgery was also associated with POAF development in the univariable logistic regression analysis, predominantly driven by combined CABG plus valvular surgery (Wald = 4.402, OR = 1.976, 95% CI = [0.046–3.732], *p* = 0.036). However, only cardiopulmonary bypass time and cancer proved to be independent predictors of POAF in the multivariable logistic regression analysis (Table 3).

**Figure 2:**
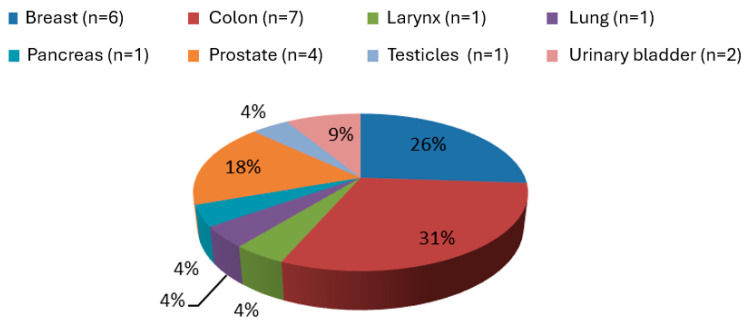
Distribution of cancer types in the study population.

The cancer types and their distribution in the study patients are presented in Figure 2. The average age was 72 [64–76] years, and there were 15 (65.2%) males and 8 (34.8%) females. The most common type of cancer was colon cancer followed by breast and prostate cancer. The characteristics of cancer patients in the POAF (−) and POAF (+) groups are presented in Table 4.

## 4. Discussion

The findings of the present study indicate that cardiopulmonary bypass time and cancer are independent predictors of POAF after cardiac surgery. Previous studies demonstrated that the POAF incidence rate depends on the type of cardiac operation, ranging from ≈20% after coronary artery bypass grafting (CABG) to ≥50% after surgical valve replacement [1]. POAF incidence was ≈30% after aortic surgery [13] and higher in combined valve and CABG operations [14,15]. In this study, the combination of CABG and valvular heart disease was associated with POAF in the univariable analysis but not in the multivariable analysis.

The pathophysiology of POAF after cardiac surgery is complex. Potential underlying mechanisms include myocardial injury leading to inhomogeneity of conduction and anisotropic conduction [16]; inflammation causing the loss of epicardial myocytes and changes in connexins [17,18]; autonomic influences and, in particular, sympathetic activation [19,20]; and subclinical hyperthyroidism related to an abnormal release of thyroid-stimulation hormone or T3-like peptides of the tumor [21]. The association of cardiopulmonary bypass time with POAF most likely reflects the ischemia–reperfusion sequence occurring during cardiac surgery, which may lead to myocardial injury [22]. Likewise, the emergence of cancer, as the major risk factor for POAF after cardiac surgery, is probably related to the altered atrial substrate and the high prevalence of cardiovascular risk factors in cancer patients [23].

CPB secures sufficient oxygenation and circulation during cardiac surgery. CPB management is complex as it includes several procedural aspects such as temperature, fluid balance, blood flow, pressure management, anticoagulation, and invasive monitoring during cardiac surgery [24]. It has been repeatedly demonstrated that CABG patients operated with CPB have a higher risk of developing POAF than those operated without CPB, indicating that CPB may play a role in POAF development [14,25].

The growing recognition of the link between cardiovascular diseases and cancer may stem from the combination of the aging population, improved cancer management that prolongs survival, and the introduction of novel cancer therapies that bring cardiovascular toxicity [9,26,27,28]. In this context, existing epidemiological investigations have demonstrated a high incidence of AF in cancer patients, either for a specific cancer type or treatment [29,30,31,32,33,34,35,36,37]. Further, although traditional and targeted anticancer therapies have significantly improved survival in several cancers, they have significantly increased the risk for AF, which has been recognized one of the most serious cardiovascular side effects as it both impacts treatment rendering drug reduction or discontinuation necessary and occasionally may lead to patient death. Several classes of anticancer therapies, including radiotherapy, have been associated with AF.

Both cancer and anticancer therapies may induce atrial structural and electrical remodeling, which underlie the development of AF. Atrial structural remodeling has been attributed to several effects including myocardial injury, inflammation, fibrosis, anemia, reactive oxygen species [ROS], hypercoagulability, and endothelial dysfunction, acting on top on traditional cardiovascular risk factors (cardiovascular disease, diabetes, obesity, and age) [38]. Atrial electrical remodeling includes increases in cytosolic Ca^2+^ levels providing an arrhythmogenic AF substrate through early and late afterdepolarizations, respectively [39], as well as the reduction and redistribution of connexin [40].

The number of cancer patients that developed POAF in this study was small enough to allow for associations to be made with cancer type (n = 10; 3 with colon cancer, 3 with breast cancer, 2 with prostate cancer, 2 with colon cancer, and 1 with larynx cancer). However, our findings are in accordance with those of recent studies indicating that the types of cancer that are more likely to be diagnosed after AF are in line with the most frequent cancer types in the general population [41]. It is noteworthy that chemotherapeutic agents, radiotherapy, and trastuzumab have been equally associated with cardiotoxicity [42,43].

## 5. Clinical Implications

The findings of this study provide further evidence supporting moving from a “one-size-fits-all” approach to a “patient-centered” approach, tailoring POAF management according to each patient’s specific features, which at the same time may also reduce the overtreatment of patients who will likely convert spontaneously to sinus rhythm-reducing cardioversion-related complications [44]. However, the risk of POAF after cardiac surgery in cancer patients is high and necessitates increased awareness and implementation of preventive measures. Many patients undergoing cardiac surgery are already on beta-blockers, and it is generally recommended to continue their use until surgery. It is less clear, however, whether beta-blockers should be administered preoperatively to patients not taking these medications. Preoperative amiodarone is the single most powerful preoperative intervention for reducing POAF risk. Several amiodarone administration protocols, both oral and intravenous, have been successfully used. In a recent study, amiodarone given within the first 24 h of admission to the cardiac surgery intensive care unit (CSICU) (iv loading dose of amiodarone 150 mg iv over one hour, followed by an amiodarone iv infusion at 1 mg·min^−1^ for six hours, then a 0.5 mg·min^−1^ iv infusion for 18 h). After the 18 h iv loading regimen, oral amiodarone 200 mg bid was prescribed for seven days (or until hospital discharge), which significantly decreased the risk for incident POAF with minor side effects [45]. A recent meta-analysis including 85 trials and 18,981 patients examined the effectiveness of anti-inflammatory agents in preventing atrial fibrillation after cardiac surgery [46]. The use of nonsteroidal anti-inflammatory drugs and statins reduced the risk of POAF compared with the placebo, both with a moderate certainty level. The use of fish oil in combination with vitamins C and E, corticosteroids, and N-acetylcysteine reduced the risk of POAF, all with a low level of certainty. All the interventions had no significant impact on mortality rate or risk of serious adverse effects [46]. Other POAF-preventing interventions include posterior pericardiotomy [47] or dual atrial chamber pacing within the first 24 h of admission to the CSICU [48].

## 6. Strengths and Limitations

The strength of this study is that it highlighted the importance of cancer as a major risk factor for the development of POAF after cardiac surgery. This study, however, has several limitations. Due to the small number of cancer patients enrolled, the features predisposing cancer patient to POAF could not be evaluated. Along the same lines, it could not be concluded with certainty which types of cancer are related to POAF development after cardiac surgery. However, the results of this study were driven by colon (n = 7), breast (n = 6), and prostate (n = 4) cancers, which accounted for 74% (17/23) of cancer cases. Finally, this study cannot answer the question of whether patients who develop POAF after cardiac surgery should be screened for cancer. However, cancer was present in 10 out of the 66 patients (15.2%) who developed POAF, and this percentage is not negligible.

## 7. Conclusions

Cancer was associated with a dramatic increase in the risk of POAF following elective cardiac surgery in the present study. The implementation of perioperative measures to prevent POAF in this patient population is required (see graphical abstract). Further studies should be conducted to clarify the futures of cancer patients as well as the types of cancer associated with the development of POAF after cardiac surgery. Likewise, whether patients developing POAF after cardiac surgery should be searched for cancer deserves further investigation.

## Figures and Tables

**Table 1:** Characteristics of the study population.

Variables	No Atrial Fibrillation (n = 334)	Atrial Fibrillation (n = 66)	*p*
**Demographic**			
Age (years)	67 [59–73]	71 [66–75]	0.200
Females (%)	48 (14.4)	16 (24.2)	0.929
**Clinical**			
Systolic blood pressure (mmHg)	130 [115–144]	130 [115–150]	0.088
Diastolic blood pressure (mmHg)	74 [65–81]	63 [71, 72]	0.698
Heart rate (bpm)	73 [65–80]	70 [65–80]	0.318
**Left ventricular ejection fraction (%)**	**58 [55–63]**	**60 [55–65]**	**0.009**
**Laboratory**			
Hemoglobin (mg/dL)	13 [11.8–14.4]	12.95 [11.7–14.9]	0.561
Hematocrit (%)	39.9 [37.2–42.3]	39.2 [36.1–42.6]	0.495
Creatinine (mg/dL)	0.93 [0.80–1.14]	0.92 [0.74–1.13]	0.978
Sodium (meq/L)	140 [139–141]	140 [139–141]	0.989
Glucose (mg/dL)	112 [101–136]	118 [101–138]	0.493
Potassium (meq/L)	3.72 [3.57–3.90]	3.79 [3.59–3.90]	0.840
**Cardiopulmonary bypass time (min)**	**126 [97–164]**	**149 [118–189]**	**0.002**
Cross clamp time (min)	74 [55–117]	92 [62–129]	0.160
**Coexisting morbidities**			
**Valvular heart disease (%)**	**155 (46)**	**41 (62)**	**0.009**
Heart failure (%)	23 (7)	3 (5)	0.518
Coronary artery disease (%)	214 (64)	35 (53)	0.164
Hypertension (%)	130 (39)	18 (27)	0.105
**Cancer (%)**	**13 (4)**	**10 (15)**	**<0.001**
Chronic obstructive pulmonary disease (%)	111 (33)	20 (30)	0.769
**Dyslipidemia (%)**	**207 (62)**	**30 (45)**	**0.026**
Hyperthyroidism (%)	4 (1)	0 (0)	0.380
Chronic kidney disease (%)	35 (10)	7 (11)	0.907
Diabetes mellitus (%)	60 (18)	8 (12)	0.292
Obesity (%)	52 (16)	8 (12)	0.535

**Table 2:** Results of univariable logistic regression analysis (dependent variable = postoperative atrial fibrillation).

Independent Variables	Wald	Odds Ratio	Lower 95% CI	Upper 95% CI	*p* Value
**LVEF**	3.483	40.458	0.830	1971.388	0.062
**CBP**	6.582	1.001	1.000	1.001	**0.010**
**VHD**	6.628	2.070	1.190	3.602	0.062
**Cancer**	11.666	4.587	1.915	10.9.85	**<0.001**
**Dyslipidemia**	4.869	0.546	0.319	0.935	**0.027**

LVEF, left ventricular ejection fraction; CBP, cardiopulmonary bypass time; VHD, valvular heart disease; CI, confidence interval.

**Table 3:** Results of multivariable logistic regression analysis (dependent variable = postoperative atrial fibrillation).

Independent Variable	Wald	Odds Ratio	Lower 95% CI	Upper 95% CI	*p* Value
Surgery type 1	3.134	0.173	0.025	1.207	0.077
Surgery type 2	1.966	0.245	0.034	1.750	0.161
Surgery type 3	2.357	0.223	0.033	1.515	0.125
Surgery type 4	<0.001	<0.001	<0.001	.	0.999
LVEF	3.654	47.976	0.907	2538.745	0.056
**CBP**	**4.671**	**1.001**	**1.000**	**1.001**	**0.031**
VHD	2.584	4.064	0.735	22.463	0.108
**Cancer**	**8.257**	**3.852**	**1.535**	**9.664**	**0.004**
Dyslipidemia	0.840	0.646	0.254	1.645	0.360

Surgery type 1, coronary artery bypass grafting (CABG); surgery type 2, CABG combined with valvular heart surgery; surgery type 3, valvular heart surgery; surgery type 4, aortic surgery; LVEF, left ventricular ejection fraction; CBP, cardiopulmonary bypass time; VHD, valvular heart disease; CI, confidence interval.

**Table 4:** Characteristics of patients with cancer.

Patient	Sex	Age	Cancer Type
**POAF (−)**			
1	Female	79	Breast
2	Female	76	Colon
3	Male	70	Pancreatic
4	Male	66	Urinary Bladder
5	Male	82	Prostate
6	Female	63	Colon
7	Male	79	Colon
8	Male	74	Prostate
9	Male	56	Colon
10	Male	60	Urinary Bladder
11	Female	62	Breast
12	Male	64	Testicular
13	Female	59	Breast
**POAF (+)**			
1	Male	71	Prostate
2	Male	76	Colon
3	Male	71	Colon
4	Female	76	Breast
5	Male	75	Laryngeal
6	Female	66	Breast
7	Male	73	Lung
8	Male	74	Prostate
9	Male	73	Breast
10	Female	72	Colon

## Data Availability

Data are contained within this article.
